# Roles of Organic Fragments in Redirecting Crystal/Molecular Structures of Inorganic–Organic Hybrids Based on Lacunary Keggin-Type Polyoxometalates

**DOI:** 10.3390/molecules26195994

**Published:** 2021-10-02

**Authors:** Ruhollah Khajavian, Vida Jodaian, Fatemeh Taghipour, Joel T. Mague, Masoud Mirzaei

**Affiliations:** 1 Department of Chemistry, Faculty of Science, Ferdowsi University of Mashhad, Mashhad 9177948974, Iran; ruhollah_khajavian2002@yahoo.com (R.K.); fatemetaghipoor@gmail.com (F.T.); 2 Department of Chemistry, Islamshahr Branch, Islamic Azad University, Islamshahr 3317843154, Iran; 3 Department of Chemistry, Tulane University, New Orleans, LA 70118, USA; joelt@tulane.edu

**Keywords:** polyoxometalates, Keggin, inorganic–organic hybrids, rare earth, lacunary

## Abstract

Lacunary polyoxometalates (LPOMs) are key precursors for the synthesis of functional POMs. To date, reviews dedicated to behavioral studies of LPOMs often comprise the role of metal ions, including transition metal (TM) and rare earth (RE) ions, in extending and stability of high-nuclearity clusters. In contrast, the role of organic ligands in the structures and properties of lacunary-based hybrids has remained less explored. In this review, we focus on the role of organic fragments in the self-assembling process of POM-based architectures and discuss relationships between the nature and structure of organic ligand and properties such as the topology of hybrid inorganic–organic material in RE and TM-RE heterometallic derivatives of lacunary Keggin-type POMs. The effects of organic fragment in mixed ligand hybrids are also briefly reviewed.

## 1. Introduction

**Figure 1:**
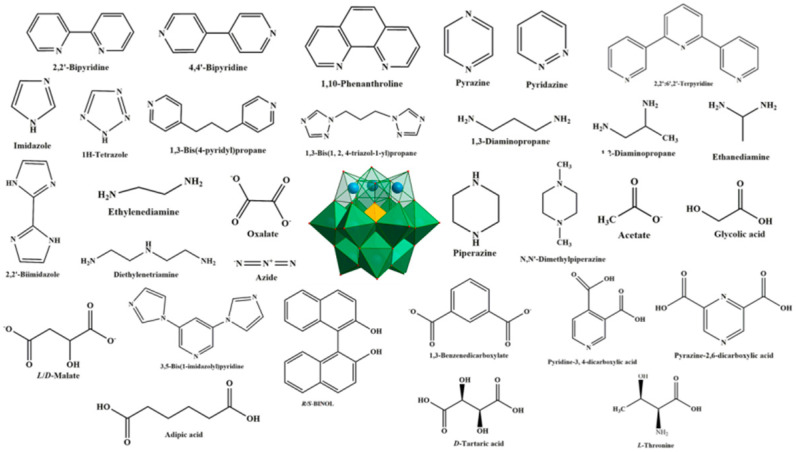
Some organic ligands employed to inorganic–organic hybrids based on lacunary Keggin-type polyoxometalates in the literature.

Polyoxometalates (POMs), a large group of fascinating polynuclear metal-oxo clusters of early transition metals such as Mo, W and V, constitute a marvelous class of inorganic systems, due to their intriguing structures and remarkable potential applications in electrochemistry, catalysis, magnetism, and medicine [1,2,3,4,5,6]. Lacunary POMs (LPOMs) with a set of remarkable properties such as high coordination reactivity, rigidity, oxidative and thermal stability are an important sub-class of POMs [7]. Typically, LPOMs imply that topologies gain by the loss of one single {MO} moiety or multiple {M_x_O_2x_} moieties, resulting in the formation of the monolacunary or polylacunary POMs, respectively. Mainly, lacunary species are limited to polyoxotungstates, while lacunary POMs of polyoxomolybdates and polyoxovanadates are uncommon [8]. Tungsten skeleton of the POMs with saturated Keggin [XW_12_O_40_]^n−^ and Wells–Dawson-type [X_2_W_18_O_62_]^n−^ structures can be partially decomposed by the addition of a weak base, while retaining the original Keggin- and Wells–Dawson-type structures, to form lacunary species, such as [XW_11_O_39_]^n−^ and [X_2_W_17_O_61_]^n−^ [9]. The lacunary species with high negative charge and nucleophilic oxygen-enriched surfaces can interact with various cations. In fact, owing to the negative-charged surface and defect binding sites, LPOMs can act as outstanding multidentate nucleophilic ligands toward the electrophilic center. Transition metal (TM) or lanthanoide (Ln) cations can be incorporated into the defect sites of LPOMs to form metal-substituted POMs, which exhibit unique chemical properties that depend on the incorporated metal ions [10,11,12,13,14,15,16,17,18,19,20,21,22]. Metal-substituted POMs possess a higher negative charge density than the saturated parent POMs due to the substitution of a high oxidation state W^6+^ ion by a low oxidation state M^n+^ ion (usually *n* = 1–3). One of the promising approaches toward the synthesis of this kind of material is combining metal-substituted POMs with organic or metal–organic fragments to make inorganic–organic hybrid-based LPOMs. Various POM-based inorganic–organic hybrids with interesting structural and functional properties were reported [23,24,25]. In this field, a certified fact is that the synthesis of such hybrids depends on the selection of POMs as inorganic building blocks and organic ligands as structure-directing and functional components. Therefore, the careful choice of POM species and organic ligands are vital for the synthesis of hybrids with intriguing topologies and improved properties. The utilization of organic fragments as a solvent or coordinated with metal centers in the metal-substituted POM compounds can improve physical and chemical properties of these compounds. Most metal-substituted POM hybrids contain N-donor organic ligands. In these structures, organic fragments not only act as charge-compensation cations, but also as multidentate chelating agents to coordinate TM or Ln cations. This highlights the significant role of organic ligands as stabilizing agents in forming hybrid structures. The general structures discussed in this review are summarized in Figure 1.

In the present review, we highlight the most important investigations of inorganic–organic hybrid compounds combining LPOMs with TM and Ln centers. This field of research reveals a great variety of N/O-donor ligands and their potential roles in the assembly of metal-substituted POMs that have not been previously reviewed. Therefore, this review provides a comprehensive description of the different roles of various ligands in their interaction with these relevant inorganic clusters. 

## 2. Inorganic–Organic Hybrids Based on TMSPs

TM-substituted polyoxometalates (TMSPs) are an important class of materials [26,27]. Compared to nonlacunary heteropolyoxometalates, the localized surface charge of LPOMs have made them reactive building blocks for the construction of these hybrids. They are commonly used as excellent precursors, since (i) the LPOMs are easy to obtain from intact materials in high yield, (ii) vacant sites are conducive to encapsulate various transition metals, including mixed-valent metal ions, and (iii) the robustness of POMs makes it possible to predict the frameworks of the ultimate products. Hundreds of different types of LPOMs saturated by TMs or even TM complexes have been reported in recent years [28,29,30,31]. However, the introduction of an organic ligand in TMSPs has a pivotal impact on the chemical/physical properties or the structural features of the hybrid via synergetic effects between the properties of the POM and those of the ligand. Therefore, the role of the organic ligands as structure-directing agents is predominant in these inorganic–organic hybrid constructions, a point that is less considered in the compounds containing polyoxometalate clusters. Because of the nucleophilic interaction between POM anions and TM complexes, popular series of inorganic–organic hybrids based on lacunary POMs are composed of discrete structures that TM complexes substituted in the vacant sites of POMs. Many structures are reported that have these characteristics [32,33,34,35,36,37]. Abundant sandwich-type TMSPs are reported to have a varied nuclear core. Among them are some TM cores that are coordinated to organic ligands. Furthermore, additional TM complexes can have roles of charge compensation or structure-directing and promote the dimension of these TMSPs. For instance, organic solvents such as DMF and DMSO can be appropriate ligands for coordination to the TMSPs [38,39,40,41]. Table 1 provides an exhaustive list of various N-/O-donor ligands that can be connected to LPOMs.

### 2.1. TMSPs with N-Donor Ligands

Metal complexes of lacuna are popular in studies of POM hybrids. For example, Kato et al. studied the interaction of some TM-substituted α-Keggin polyoxotungstates with cis-platinum(II) moieties containing N-donor ligands [42,43,44]. Magnetic properties are highly dependent on the nature of the bridging ligands. In the KRb_5_[(PW_10_O_37_)(Ni(H_2_O))_2_(*μ*-1,1-N_3_)]·19H_2_O discrete cluster, N_3_^−^ is connected with two Ni^II^ ions substituted in the vacant sites of the polyanion and the relationship between the structural parameters and the value of the magnetic exchange parameter J for *μ*-1,1-N_3_ complexes is considered [45].

Multi-dentate N-donor ligands as structure-stabilizing agents can capture and stabilize the in situ-generated TM oligomers or aggregates to construct novel POMs. en is another bidentate N-donor organic fragment that has largely been employed in the TMSPs. Compounds such as [Ni_6_(*μ*_3_-OH)_3_(en)_3_(H_2_O)_6_(B-*α*-AsW_9_O_34_)]‧6H_2_O and [Ni_6_(*μ*_3_-OH)_3_(en)_3_(H_2_O)_6_(B-*α*-AsW_9_O_34_)]‧10H_2_O are important examples of hexa-Ni-substituted POMs based on single lacunary Keggin fragments because they contain the highest number of transition metal ions in known lacunary Keggin *arsenotungstate* monomers [46,47]. Discrete dimers (H_2_en)_2_[Cu^II^_8_(en)_4_(H_2_O)_2_(B-*α*-GeW_9_O_34_)_2_]‧5H_2_O and (H_2_en)_2_[Cu^II^_8_(en)_4_(H_2_O)_2_(B-*α*-SiW_9_O_34_)_2_]‧8H_2_O are constructed from two trivacant Keggin [B-*α*-XW_9_O_34_]^10−^(X=Ge^IV^/Si^IV^) fragments and an octa-Cu cluster [48]. In these structures, hydrogen bonding interactions between the nitrogen atoms of en ligands and surface oxygen atoms of polyoxoanions generate extended supramolecular networks [49]. In the case of H_10_K_2_Na_2_[Zr_4_(*μ*_3_-O)_2_(*μ*-OH)_2_(en)_2_(B-*α*-GeW_10_O_37_)_2_]‧4DMF‧22H_2_O as the first Zr-substituted POM with N–Zr–N bonds; however, a 1D chain structure is formed through the connection of potassium cations and sandwich-type polyoxoanion [50]. Another interesting example is the structure of [Cu(en)_2_(H_2_O)]_2_{[Cu(en)_2_][*α*-PCuW_11_O_39_Cl]}‧3H_2_O, which is similar to those of {[Cu(en)_2_(H_2_O)][Cu(en)_2_]_2_[*α*-PCuW_11_O_39_Cl]}‧6H_2_O and [{Cu(en)_2_}_3_(*α*-PCuW_11_O_39_Cl)]‧6H_2_O [51]. However, the first is an isolated structure whereas the second and third are 1D linear polymeric chains through [Cu(en)_2_]^2+^ bridges via Cu–Cl–Cu–O–W linkages [52]. Belt-like hexa-Cu^II^ cluster-substituted POM, [Cu_6_(en)_2_(H_2_O)_2_(B-*α*-GeW_9_O_34_)_2_], in the presence of two [Cu(en)_2_]^2+^ units or [Cu_4_(deta)(H_2_O)]^2+^ complex bridges form a 1D chain structure, as is found in the other similar hexa-Cu^II^ cluster-substituted POM with Cu(en)_2_ bridges [53,54]. In these cases, the coordination of en ligands on the hexa-CuII cluster increases the number of the TM cations in the sandwich belt. In fact, each [Cu_6_(en)_2_(H_2_O)_2_(B-*α*-GeW_9_O_34_)_2_] as a complete cluster connects four others via four [Cu(en)_2_]^2+^ bridges to form a novel 3D framework, which is rare in sandwich-type POMs [55,56]. Similarly, [Cu^II^(H_2_O)_2_]_2_[Cu^II^_8_(en)_4_(H_2_O)_2_(B-*α*-SiW_9_O_34_)_2_] displays 3D (3,6)-connected nets with (4‧6^2^)-(4^2^‧6^4^‧8^7^‧10^2^) topology, which are built by octa-Cu sandwiched polyoxometalate building blocks through copper cation bridges [48].

[{Cu_6_(*μ*_3_-OH)_3_(en)_3_(H_2_O)_3_}(B-*α*-PW_9_O_34_)]·7H_2_O exhibits an unprecedented 3D framework with hexagonal channels enclosed by three interweaved helical chains in the TMSP chemistry [46]. The steric effect of organic ligands is known to be an important factor in the modification of the structure of coordination compounds, and smaller steric hindrance of the organic ligands favored the POM anions functioning as high-connected linkages in coordination compounds. Thus, the substitution of dap by en organic ligands in the compounds with the same dimeric Keggin polyoxoanions [(PW_11_CuO_39_)_2_]^10−^ results in different dimensionality. [Cu(en)_2_]^2+^ fragments have smaller volume and steric hindrance in comparison with Cu(dap)_2_ and, hence, have easy access to [(PW_11_CuO_39_)_2_]^10−^ dimers to link with their external active oxygen atoms, forming higher-dimensional and higher-connected POM-based hybrid compounds [52,57]. 

Sandwich-type structures can encapsulate TM clusters. {[Cu(dap)_2_(H_2_O)]_2_[Cu_6_(dap)_2_(B-*α*-SiW_9_O_34_)_2_]}‧4H_2_O is an example of a sandwich-type structure with a hexa-nuclear ring [58]. Some discrete dimers are also constructed from two trivacant Keggin [B-*α*-XW_9_O_34_]^10−^(X=Ge^IV^/Si^IV^) fragments and a {Cu_8_(dap)_4_} complex [46,48]. The double-cluster complex of [{Ni_7_(*μ*_3_-OH)_3_O_2_(dap)_3_(H_2_O)_6_}(B-*α*-PW_9_O_34_)][{Ni_6_(*μ*_3_-OH)_3_(dap)_3_(H_2_O)_6_}(B-*α*-PW_9_O_34_)][Ni(dap)_2_(H_2_O)_2_]·4.5H_2_O is a unique structure containing both hexa- and hepta-Ni^II^-substituted trivacant Keggin clusters of {Ni_6_(dap)_3_PW_9_} and {Ni_7_(dap)_3_PW_9_} [46]. Linear organic ligands such as dap can link the sandwich POTs as nodes [59,60]. For example, Chen et al. reported a tetra-nuclear {Co^II^_4_(Hdap)_2_} substituted sandwich-type Keggin germanotungstate unit with [Co(dap)_2_]^2+^ bridges that represented the first 2D organic–inorganic hybrid cobalt-substituted sandwich-type polyoxotungstate [61]. 

[Ni_6_(*μ*_3_-OH)_3_(H_2_O)_2_(dien)_3_(B-*α*-PW_9_O_34_)].4H_2_O is the first extended TMSP with a 1D zigzag chain made of Ni and PW_9_O_34_ SBUs via corner-sharing between {NiO_6_} and {WO_6_} octahedra via intermolecular interactions. This compound contains the highest number of Ni ions in any known lacunary Keggin polyoxotungstate, as well [62]. In addition to its usual coordination mode, dien can bridge between metal centers, as well. For example, a 2D layer-like structure has been recently reported in which [Bi_2_W_20_O_70_]^14−^ polyanions are linked by [Cu_2_(dien)_2_]^4+^ bridges [63].

bpyand phen, as rigid organic ligands, are powerful tools controlling the structure of the final product because they can act as structure-stabilizing agents or structure-directing agents to influence coordination geometries or connecting modes of transition-metals in the vacant sites of POTs in the construction of novel TMSPs. In addition, the N-donor groups may also generate hydrogen bonding interactions and π–π interactions in the formation of target structures, which may affect the properties of complexes [64,65,66,67,68,69]. A survey of the literature shows that investigations on bpy/phen-containing copper-complex-substituted Keggin POTs have been reported extensively [70,71]; however, organic–inorganic hybrid di-copper-bpy/phen-substituted monovacant Keggin POTs have been explored to a limited extent.

Compound [Cu^I^_3_(phen)_3_Cl_2_][Cu^II^(phen)_2_][Cu^II^_2_(phen)_2_(*α*-PW_11_O_39_)] represents the first discrete di-copper-phen complex-substituted monovacant Keggin polyoxotungstate. It is of note that the π–π interactions between adjacent phen rings may play an important role in the structure stabilization of the title compound [43,68]. In the compound [Cu^I^_3_(pz)_2_(phen)_3_]_2_[Cu^I^(phen)_2_][{Na(H_2_O)_2_}{(V^IV^_5_Cu^II^O_6_)(As^III^W_9_O_33_)_2_}]·6H_2_O, two {AsW_9_O_33_} clusters are connected by the mixed hexa-TM ring unit {V^IV^_5_Cu^II^O_6_} to form a sandwich-type dimer, which are further bonded in “ABAB” mode by the {Na(H_2_O)_2_} linker, resulting in pure inorganic chains. The unique “L-shaped” trinuclear complex {Cu_3_(pz)_2_(phen)_3_} is supported together via staggered π–π interactions to generate extending waveform 2D supramolecular layers, which are further aggregated with their adjacent analogues by complexes {Cu(phen)_2_} via H-bonding interactions to yield an unprecedented 3D metal–organic network with 1D cavities. The pure inorganic 1D sandwich chains are implanted in the cavities as guest units via supramolecular interactions to form a POMOF 3D framework [72]. In the skeleton of [Cu^II^(bpy)(H_2_O)][Cu^II^_2_(bpy)_2_(H_2_O)(*α*-HPW_11_O_39_)]·H_2_O, there are three crystallographically unique Cu^II^ cations. They all adopt the square pyramid geometry with two N atoms from 2,2′-bpy ligands and three O atoms from the [*α*-PW_11_O_39_]^7−^ fragments or water molecules. This complex displays a 1D zigzag infinite chain architecture constructed by alternating di-copper-bpy-substituted [Cu^II^_2_(bpy)_2_(H_2_O)(*α*-HPW_11_O_39_)] polyoxoanions with Cu–O linkers. Notably, this compound is a new di-transition metal-bpy-substituted monovacant Keggin phosphotungstate with a 1D dual-bridging chain structure [68]. 

The reactivity of other nitrogen heterocyclic ligands such as imi has also been studied. It has been shown that imi ligands connect to metal ions via M–N_imi_ bonds, and no M–C_imi_ bond in POMs has been observed to date [73,74,75,76,77]. In addition, numerous studies have shown that imi could interfere with DNA via weak interactions (hydrogen bonds, π–π stacking, etc.), and then halt cell growth and division [78,79]. Compound {[Ag_7_(H_2_biim)_5_][PW_11_O_39_]}·Cl·H_3_O displays a 2D network featuring dimerized monolacunary Keggin anions {PW_11_O_39_}_2_ which are connected through hexanuclear silver clusters. Interestingly, besides {Ag_5_}^5+^ clusters, there are other kinds of argentophilic {Ag_4_}^4+^ clusters coexisting in this compound [80]. Liu et al. reported three hexa-nuclear-substituted sandwich-type arsenotungstates [81]. The transition metal ions (Ni^II^, Co^II^, and Mn^II^) and Na^+^ are alternately coordinated in the six-membered central belt by [*α*-AsW_9_O_33_]^9−^ units, taz, and water molecules. The contribution of nitrogen atoms of the ligands in the formation of hydrogen bonding network leads to the fortification of the structures [81]. Htz as a rigid multifunctional ligand can provide four sequent electron-donating nitrogen atoms to coordinate to metals with the smaller steric hindrance. Many metal–organic frameworks based on Htz ligands exhibit intriguing topologies and interesting magnetic, absorptive, and photophysical properties, having diverse coordination/bridging modes [82,83]. For example, in {[Cu_8_(tz)_8_(Htz)_4_(H_2_O)_5_][PMo^VI^_10_Mo^V^O_39_]}∙~10H_2_O, the six-nuclear copper clusters are bridged by the tz ligands to form wave-like layers by the *μ*_2_-Htz ligands. The polyoxomolybdate anions act as the eight-connected node to link the layers into a 3D framework [84].

### 2.2. TMSPs with O-Donor Ligands

O-donor ligands have been comparatively less studied than flexible nitrogen donating ligands for the design and synthesis of new hybrids. As a peculiar branch of POMs, considerable attention has been directed toward POM-based metal carbonyl derivatives in recent years because of their unique structures and potential catalytic properties [85,86,87,88]. For example, Na_2_H_2_[(CH_3_)_4_N]_6_[Te_2_W_20_O_70_{Re(CO)_3_}_2_]·20H_2_O is a monomeric tellurotungstate(IV)-supported rhenium carbonyl derivative similar to that of the previously reported tricarbonyl metal polyoxoanion complexes [X_2_W_20_O_70_{M(CO)_3_}_2_]^12−^ (X = Sb, Bi and M = Re, Mn) composed of two identical {B-*β*-TeW_9_O_33_} units joined by two {WO_6_} octahedra. Furthermore, two Re atoms and two central W atoms are located in the same plane and each carbonyl rhenium group fac-{Re(CO)_3_}^+^ is in the “out-of-pocket” structural motif [88].

Acetate has been frequently used as a bridging ligand to connect different fragments. For example, in the monomeric structure of [*γ*-H_2_SiW_10_O_36_Pd_2_(OAc)_2_]^4−^, Pd atoms are bridged by two bidentate acetate ligands [89]. However, the synthesis is usually performed in acetate buffer solution [90]. Crown-shaped Ru-substituted arsenotungstate [As_4_W_40_O_140_{Ru_2_(OAc)}_2_]·22H_2_O present a new acetate-bridged Ru-substituted arsenotungstate. [As_4_W_40_O_140_]^28−^ cyclic unit embeds two [Ru_2_(OAc)]^7+^ segments in its cavities. A bidentate acetate ligand connects two diametrically opposed Ru atoms in a (*μ*_2_-η^1^:η^1^) fashion. As far as we know, such a crown-shaped acetate-bridged Ru-substituted arsenotungstate is the first report that supports the structural novelty of this rare compound [91]. Kholdeeva, Kortz, and co-workers reported isolated polyanions with unprecedented hexa-zirconium and hexa-hafnium core and the metal ions occupying the vertices of an octahedron that is accommodated by two (B-*α*-AsW_9_O_33_) fragments. The two {AsW_9_} units are not eclipsed, leaving a cavity perfectly suitable to host the M_6_ unit. Furthermore, five bridging acetate ligands lead to stronger bonding between metal centers [92]. The first carboxylic group decorated arsenotungstate was reported in 2015 [93]. Each Mn^II^ ion in the Na_15_[(Mn^II^(COOH))_3_(AsW_9_O_33_)_2_]‧15H_2_O is chelated with four oxygen atoms (*μ*_3_-O) from four {WO_6_} octahedra belonging to two different {AsW_9_} units and edge-sharing with two adjacent {NaO_6_} groups. The most interesting structural feature is that three carboxy groups are separately bonded to three manganese ions. 

**Figure 2:**
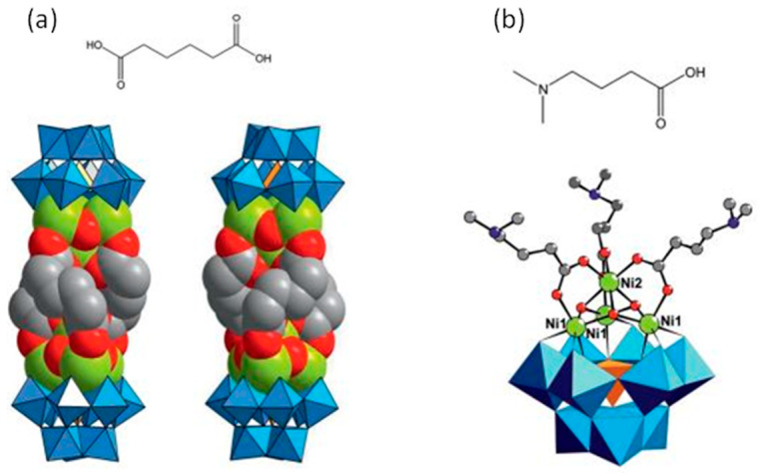
(**a**) Structure of Na_2_K_12_[Ni(H_2_O)_6_][(SiW_9_O_34_)_2_(OH)_6_Ni_8_(C_6_H_8_O_4_)_3_]·40H_2_O hybrid with adipic acid as ligand; (**b**) structure of Na_1.5_K_2.5_[Ni(H_2_O)_6_]_0.5_(SiW_9_O_34_) (OH)_3_Ni_4_(C_6_H_13_NO_2_)_3_]·17H_2_O hybrid with dimethylaminobutiric acid as ligand [96].

[Ni(en)(H_2_O)_4_]_3_[Ni_6_(en)_3_(Tris)(1,3-bdc)_1.5_(B-*α*-PW_9_O_34_)]_2_·8H_2_O represents the first 2D polyoxometalate cluster organic frameworks with honeycomb-like lattice consisting of [Ni_6_(en)_3_(Tris)(B-*α*-PW_9_O_34_)] structural building units linked by 1,3-bdc ligands. Although the honeycomb-like lattice-based POMs and organic ligands have been investigated, there is no example based on TMSPs as structural building units. It is remarkable that H[Ni_0.5_(en)(H_2_O)][Ni_6_(en)_3_(OAc)_2_(Tris)(H_2_O)_2_(B-*α*-PW_9_O_34_)]·5H_2_O is functionalized by three types of organic ligands, which is rare in POMs. Furthermore, the nitrogen atoms of the Tris ligand incorporate in hydrogen bonding and help to expand the structure [94]. Subjoining of multi-dentate organic ligands such as tartaric acid and glycolic acid into tetra-nuclear sandwich-type [Zr_4_(H_2_O)_2_(*μ*_3_-O)_2_(GeW_10_O_37_)_2_] cluster not only helps in the fortification of the sandwich-type cluster, but also, in some cases, induces chirality in the polyoxometalates [95]. Utilization of the dicarboxylato ligands such as [OOC(CH_2_)_4_COO]^2−^ (adipate), instead of monocarboxylate ligands, can lead to the oligomeric structure. As shown in the dimeric helical Na_2_K_12_[Ni(H_2_O)_6_][(SiW_9_O_34_)_2_(OH)_6_Ni_8_(C_6_H_8_O_4_)_3_]·40H_2_O structure, three adipato linkers connect two {SiW_9_Ni_4_} units (Figure 2) [96]. Liu et al. reported unique nona-Mn^II^-encapsulated sandwich-type species in which the utilization of three oxalate ligands lead to the formation of planar hexagonal {Mn_6_} core. This structure was further connected to another three external Mn^II^ cations and constructed a 1D oxalate-bridging high-nuclear Mn-sandwiched antimonotungstate chain [97]. The utilization of chiral ligands and transfer of chirality to achiral LPOM units has been reported by using L- or D-tartrate units (tart) in the large polyoxoanion compound [*α*-P_2_W_15_O_56_]^12^ [98]. In another interesting study, Ishimoto et al. successfully prepared a BINOL-functionalized lacunary Keggin-type POM for the asymmetric oxidation of thioanisole [99]. This is especially true in POMs, which contain multiple metal centers that are subject to rapid racemization via water exchange, partial hydrolysis, or fluxional rearrangements. As a result, it is often challenging to discriminate between enantiomers and/or diastereomers, and even more formidable to achieve partial or complete resolution. The polytungstate assembly of [(*α*-P_2_W_16_O_59_)Zr_2_(*μ*_3_-O)(mal)]_2_^18−^ consists of two divacant [*α*-P_2_W_16_O_59_]^12−^ anions linked by four Zr cations and is constitutionally similar to the centrosymmetric complex, [Zr_4_(*μ*_3_-O)_2_(*μ*_2_-OH)_2_(H_2_O)_4_(P_2_W_16_O_59_)_2_]^14−^, reported by Pope et al. [100]. While in it, the *μ*_2_-OH and terminal aqua ligands are replaced by the oxygens of the ligating malates (from carboxylate and hydroxo moieties) [101]. Changing organic units to D/L-mandelic acid has been seen in similar architecture [102]. Wang et al. also reported four similar chiral sandwich-type compounds consisting of tetra-Zr^IV^-substituted sandwich-type Keggin polyoxoanion and L-/D-mal fragments. The most striking structural feature of (NH_4_)_3_Na_2_K_5_[Zr_4_(μ_3_-O)_2_(L-mal)(D-mal)(B-*α*-SiW_10_O_37_)_2_] relative to similar compounds is that each [Zr_4_(*μ*_3_-O)_2_(L-mal)(D-mal)(B-*α*-SiW_10_O_37_)_2_]^10−^ polyoxoanion joins adjacent six K^+^ bridges and each K^+^ bridge links to adjacent three [Zr_4_(μ_3_-O)_2_(L-mal)(D-mal)(B-*α*-SiW_10_O_37_)_2_]^10−^ polyoxoanions, leading to a 2D layer. Furthermore, adjacent 2D layers are interconnected by two Na^+^ cations, forming a 3D framework [103].

## 3. Inorganic–Organic Hybrids Based on RESPs

It is apparent that most mentioned RE-POMs are purely inorganic, and the investigations of organic–inorganic hybrids based on RE-POMs are relatively scarce. Although the simultaneous presence of organic and LPOM ligands that are bound to RE centers is also rare, one can expect that their properties are conveyed to novel hybrid molecules [110]. Occasionally, organic solvents can be connected to metal centers in the POM-based structures. For example, solvents such as acetone, DMSO, and DMF can play the role of a ligand in the dimeric, 1D, and 2D inorganic–organic hybrids based on RESPs by coordinating to metal centers [111,112]. By considering structural features, organic ligands with their functional groups reduce distances between RE centers and facilitate the formation of polynuclear fragments. Thus, in this section, we discuss the role of these interactions in the stability and structural features of rare-earth-substituted inorganic–organic hybrids. The tendency of RE centers to carboxylate groups results in effective interactions between these fragments. Naruke and co-workers reported [Ce_3_(CO_3_)(SbW_9_O_33_)(W_5_O_18_)_3_]^18−^ anion with two different types of POMs. In the crystal structure, the triangular [Ce_3_(CO_3_)]^7+^ core was surrounded by one trivacant [B-*α*-SbW_9_O_33_]^9−^ and three monolacunary Lindqvist anions [W_5_O_18_]^6−^ [113]. The presence of carbonate in the structure led to the isolation of a compound that was isomorphous to another structure, previously reported by the authors [114].

Acetic acid has been frequently used as a bridging agent in the synthesis of inorganic–organic hybrids because of its small size and tendency to RE metals [115]. In the dimeric structures of {RE(α-XW_11_O_39_)(H_2_O)}_2_ [X = Si, Ge, P], acetic acid units act as linkers via (η^2^, *μ*-1,1) fashion, connecting two RE centers. The atomic radius of metals has an important role in the construction of such compounds (Table 2) [116,117,118,119]. When Na_9_[*B*-*α*-AsW_9_O_33_] as precursor reacts to RE ions, it is involved in decomposition and rearrangement reactions that leads to the formation of [B-*b*-AsW_9_O_33_]^−9^, {W_3_O_13_}, {W_2_O_10_}, and {WO_6_} fragments. These components are coordinated with RE cations while acetate groups act as ancillary ligands [120]. 

Amino acids, as a type of carboxyl-and-amino-containing flexible multidentate ligand, are outstanding candidates for performing as organic modifiers in the building of novel structures [121,122]. In this regard, four gly amino acids linked two {RE(*α*-BW_11_O_39_)} subunits by sharing O atoms in *μ*_2_-O or *μ*_3_-O modes. Neighboring [RE_2_(gly)_4_(*α*-BW_11_O_39_)_2_]^12−^ units were oppositely aligned in a staggered fashion in which N–H∙∙∙O hydrogen bonds between gly molecule and surface O atoms of POM units as well as Van der Waals interactions generated the 3D supramolecular framework [123]. The polyoxoanion skeletons of three kinds of _L_-ala-decorated and RE-incorporated arsenotungstate hybrids are similar. It can be designated as two [As_2_W_19_O_68_]^16−^ polyanions encapsulating an ala-decorated W‒O‒RE heterometallic cluster ([Eu_4_W_5_(H_2_O)_10_(ala)_3_O_14_]^14+^, [RE_4_W_6_(H_2_O)_8_(ala)_4_O_15_(OH)_2_]^16+^ (RE = Gd^III^, Tb^III^), and [RE_4_W_6_(H_2_O)_10_(ala)_2_O_15_(OH)_2_]^16+^ (RE = Dy^III^, Ho^III^, Er^III^, Yb^III^, Lu^III^), concluding in a four-leaf-clover-shaped tetrameric structure. However, the major inconsistency in the ala-decorated W‒O‒RE heterometallic clusters lies in the number of ala molecules, which may result from the different coordination geometries of RE ions and the various construction modes of W‒O‒RE heterometallic clusters. It should be mentioned that the carboxyl groups of ala ligands only coordinate with the W centers in the compounds containing Dy^III^, Ho^III^, Er^III^, Yb^III^, and Lu^III^ ions, and they not only link the W centers together but also combine RE ions in the other compounds [124].

Although ox ligands, such as the aforementioned ligands containing carboxylate groups, act as a linkage in the dimer of {RE(*α*-XW_11_O_39_)} subunits, their distances between RE centers are different (Table 2) [125]. In some cases, this ligand imposes polynuclear structures and deduces tetrameric moieties [126,127]. Tartaric acid compared to oxalic acid is more flexible, but their carboxylate groups can be completely or partially deprotonated. Hence, two {RE_2_(AsW_9_O_33_)} subunits can be linked by two series of non-equivalent tartaric acid segments in an unusual fashion [128]. For the dimeric polyoxotungstate [Ho(tart)(*α*-PW_11_O_39_)]_2_^16−^, the tartrate anion connects two Ho^III^ ions by one carboxyl O atom and one hydroxyl O atom from each end of the tart ligand. Furthermore, two tartrate anions displayed a type of mesomeric configuration with the co-existence of the *D*-tartrate anion and *L*-tartrate anion, which played a significant role in understanding the organic carboxylic acid functionalization. Both two nonequivalent eight-coordinate Ho^III^ centers demonstrated distorted square antiprism configurations, which are all defined by four O donors from the two tartrate fragments and four O atoms from the lacunary site of the [*α*-PW_11_O_39_]^7−^ polyanion [129]. K_11_LiH_21_[RE_3_(H_2_O)_7_{RE_2_(H_2_O)_4_As_2_W_19_O_68_(WO_2_)_2_(C_6_O_7_H_4_)_2_}_3_]∙nH_2_O (RE = Y, Tb, Dy, Ho, Er, Tm, Yb, Lu) is the first high-nuclear RE metal-substitute arsenotungstate aggregate with citric bridges. In these hybrids, the citrate fragments link RE centers and {WO_2_} units to form trimeric structures [130]. Two Dy ions occupy non-adjacent sites of lacunary {AsW_10_O_38_} polyanion, and each of them is coordinated to a tridentate citric acid ligand. Strong hydrogen bonding resulting from lattice water molecules has fortified the architecture [131]. In the dimeric structure of K_20_Li_2_[RE_3_(*µ*_3_-OH)(H_2_O)_8_(AsW_9_O_33_)(AsW_10_O_35_(mal))]_2_∙17H_2_O (RE = Dy, Tb, Gd, Eu, and Sm) a tri-RE cluster [RE_3_(*µ*_3_-OH)(H_2_O)_8_]^8+^ linked {AsW_10_O_35_(mal)} and {AsW_9_O_33_} building blocks by sharing carboxylate groups of mal ligands [132]. Structural characterization of Na_4_H_8_[{Pr(H_2_O)_2_}_2_{As_2_W_19_O_68_}{WO_2_(mal)}_2_]·24H_2_O revealed that the Pr^III^ ions formed a 1D infinite helical chain-like architecture by hinging between organo-functionalized [{As_2_W_19_O_68_}{WO_2_(mal)}_2_]^18−^ polyanions. In this case, mal ligands stabilized the structure by the formation of five-membered W–O–C–C–O chelate rings [133].

Bulky ligands containing carboxylate groups may rarely show the bridging effect [134,135]. It was observed that pyridine-4-carboxylic acid can only substitute the water molecules that were coordinated to the bridging lanthanide cation in the structure of [REK(H_2_O)_12_][RE(H_2_O)_6_]_2_[(H_2_O)_4_RE(BW_11_O_39_H)]_2_∙20H_2_O due to the to the steric encumbrance [136]. Ritchie et al. utilized middle RE metals including europium, terbium, and K_14_[As_2_W_19_O_67_(H_2_O)] units and also Hpic precursors to establish sandwich structures containing two {B-*β*-AsW_8_O_30_} subunits. A considerable point was the embedment of three {WO_2_(pic)} fragments in the [Tb_2_(pic)(H_2_O)_2_(B-*β*-AsW_8_O_30_)_2_(WO_2_(pic))_3_]^10−^ architecture. The addition of RE components (coordinated to the non-sandwiched {WO_2_(pic)} unit) led to the development of [(RE)_8_(pic)_6_(H_2_O)_22_(B-*β*-AsW_8_O_30_)_4_(WO_2_(pic))_6_]^12−^ (RE = Tb, Eu) structures [137]. In the structure of tetrameric [RE_2_(H_2_O)_4_(pic)_2_W_2_O_5_][(RE(H_2_O)W_2_(pic)_2_O_4_)(B-*β*-TeW_8_O_30_H_2_)_2_] (RE = La^III^, Ce^III^, Nd^III^, Sm^III^, Eu^III^), Hpic ligand stabilized the structure by connecting W and RE atoms together by forming stable N−O−C−O−W five-membered-rings or N−O−RE−O−W−O six-membered-ring motifs [138]. The reaction of 3,4-pdc, RE ions, and monolacunary silicotungestate polyanions lead to monomeric (3,4-pdc)_2_RE(H_2_O)_2_SiW_11_O_39_ (RE = La, Pr, Dy) structures. In these structures 3,4-pdc was coordinated to RE centers of RESPs, stabilizing the RE ions on the POM polyanion and preventing them from RE-precipitation. In addition, intermolecular interactions increased the dimensions of architecture [18]. [PW_11_O_39_RE(phen)(H_2_O)]_2_·(phen)_8_·8H_3_O (RE = Pr, Gd, Sm, La) are the first N-containing organic ligands of functionalized mono-RE-substituted polyoxometalates. These dimeric structures consist of two similar RESPs and coordination of the RE center to the surface oxygen atom of adjacent POM unit adjoins these subunits. Strong π–π interactions between phen moieties coordinated to RE centers form a novel 1D supramolecular chain structure. Other noncovalent interactions such as O−H⋯O, N−H⋯O, and π–π interactions lead to the construction of a 3D supramolecular structure [139].

Organophosphonates as multidentate ligands can provide appropriate electron-donor features and symmetry for the building of high nuclearity RESPs, offering additional stability and opportunity to fine-tune the properties. For example, in the polynuclear clusters of [Dy_6_(ampH)_4_(H_2_O)_23_(ampH_2_)(PW_11_O_39_)_2_] and [Dy_9_(CO_3_)_3_(ampH)_2_(H_2_O)_12_(PW_10_O_37_)_6_]^35−^, Dy ions were linked together by bridging oxido ligands from the ampH− ligands to form sandwich-type and propeller-shaped POMs [140].

**Figure 3:**
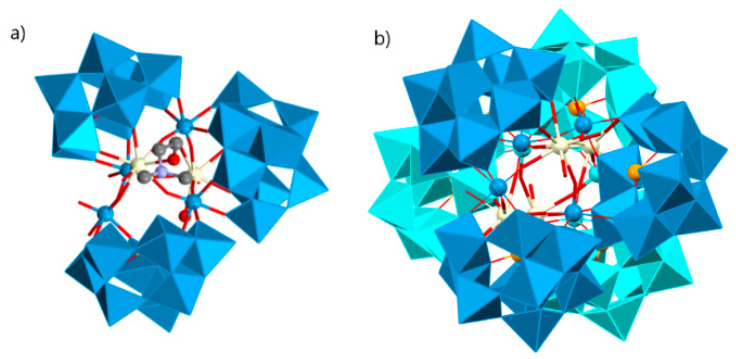
(**a**) Trimeric [Ce_2_(H_2_O)_6_(DMEA)W_4_O_9_(α-SeW_9_O_33_)_3_] cluster. (**b**) Hexameric [Ce_2_W_4_O_9_(H_2_O)_7_(α-SeW_9_O_33_)_3_]_2_ polyoxoanion structure [141].

In some cases, organic ligands not only do not contribute to the increasing nuclearity of a structure but also acts vice versa. A notable example is the work of Li et al. Because of the blocking effect of DMEA, the trimeric polyoxoanion [Ce_2_(H_2_O)_6_(DMEA)W_4_O_9_(*α*-SeW_9_O_33_)_3_] with an infrequent V-shaped [Se_3_W_29_O_103_]^20−^ group was obtained (Figure 3a), whereas in the presence of DMAHC, the hexameric polyoxoanion [Ce_2_W_4_O_9_(H_2_O)_7_(*α*-SeW_9_O_33_)_3_]_2_^24−^ was constructed from two equivalent trimeric subunits through two −O−W−O−Ce−O− connections (Figure 3b) [141].

## 4. Inorganic–Organic Hybrids Based on PBTREHDs

In recent years, the design and synthesis of 3d-4f inorganic–organic hybrids based on RESPs has increasingly become an emerging field of research due to an undeniable competitive reaction among strongly oxyphilic RE cations and less active TM cations by highly negative POM precursors in the reaction system. Thus, the discovery and recognition of novel POM-based TM–RE heterometallic derivatives with remarkable structures and properties remains a severe and inquisitorial challenge [142,143,144,145]. Such architectures containing TM and RE centers can lead to the formation of discrete to 3D nets. In some cases, N-donor organic fragments are connected to TMs rather than RE metal centers. These TM complexes can be seen as charge-balancing cation, linkage, and directing agents for the construction of high dimension architectures. In the simplest case, complexes of TMs only act as charge-balancing agents for RESPs, such as discrete structures of 1:2 sandwich-type {Dy(GeW_11_O_39_)_2_} subunits that are decorated by [Cu(H_2_O)_3_]^2+^ and [Cu(H_2_en)(Hen)]^5+^ coordination cations. These Cu metal centers decrease the negative charge over the heteropolyanions to stabilize the whole framework [146]. In another work, two lacunary [*B-α*-GeW_9_O_34_]^10−^ Keggin moieties were linked together via a rhomb-like {Ce^IV^Cu^II^_3_O_18_} cluster to form a Weakley-type structure [147]. Compared to the TM-substituted germanotungstate [148], the substitution of one external copper ion by a {Ce(OAc)}^3+^ group led to the increase in Cu···Cu distance [147].

Dolbecq, Mialane, and co-workers reported heterometallic cubane clusters of three monolacunary silicotungstates based on {LnCu_3_(OH)_3_O} (Ln = La, Gd, Eu) units [149]. It was observed that the presence of exogenous ligands is essential for obtaining such structures. In addition, the radius of the rare earth center played a crucial role in the dimensionality of the isolated compound [149]. In a similar manner, Wu et al. obtained {DyMn_4_} cubanes sandwiched by two tetravacant silicotungstates [150]. Other appended cubanes {REMn^III^_4_} (RE = Ho^III^, Tm^III^, Yb^III^, Sm^III^, Gd^III^, Er^III^, and Ce^IV^) were also reported using tetravacant {SiW_8_O_31_} ligand [151].

Trilacunary {SbW_9_O_33_} can effectively assemble RE ions and TMs into aggregates in the presence of different anions. The resulting compounds K_5_Na_11_[RE_3_(H_2_O)_3_Ni^II^_3_(H_2_O)_6_(SbW_9_O_33_)_3_(WO_4_)(CO_3_)]·(H_2_O)_40_ (RE = La^III^, Pr^III^, and Nd^III^) exhibited cyclic trimeric aggregates of three {SbW_9_O_33_} units enveloping one CO_3_^2−^-templated and one WO_4_^2−^-templated trigonal-prismatic {RE_3_(H_2_O)_3_Ni^II^_3_(H_2_O)_6_(WO_4_)(CO_3_)} units [152]. When complexes of TM act as linking centers between RESPs, they can establish high-dimension architectures. The coordination bond interactions and weak interactions between adjacent 1D chains play a significant role in the zigzagging distances and angles of different 1D chains. Numerous structures were reported in which [Cu(en)_2_]^2+^ moieties connected to well-known {RE(XW_11_O_39_)_2_} (X = P, Si, Ge) dimeric structures. For the dimeric structure of [RE(PW_11_O_39_)_2_] (RE = La, Ce, Pr, Nd, Sm, Er), 1D zigzag chain structures (*via* Cu(en)_2_ linkage) were extended to 2D sheet structures by weak interactions between the 1D chain and [Na_2_(en)_2_(H_2_O)_5_]^2+^ linking cluster presented in other zigzag chain structures. These resemblance chains were different in bonding distances and angles, due to the coordination bond and weak interactions between adjacent 1D chains [153,154]. {Cu(en)_2_} unit in the [Cu(en)_2_]_4_[RE(PW_11_O_39_)_2_]_2_^14−^ (RE = Ce, Pr) contributed to the generation of 1D zigzag chains which was further extended to 2D supramolecular architectures through hydrogen bonding between nitrogen atoms and [Ce(PW_11_O_39_)_2_]^11−^ polyanions of the neighboring 1D chain [155]. In the case of H_8_[Cu(en)_2_H_2_O]_4_[Cu(en)_2_]{[Cu(en)_2_][Pr(PW_11_O_39_)_2_]}_2_∙2en∙12H_2_O, however, weak Cu∙∙∙O electrostatic interactions contributed to making a bidimensional sheet structure [155]. In addition to {Cu(en)_2_} complexes [156,157,158,159], the presentation of other metal centers such as K^+^ ions were reported to help construct a 1D chain consisting of [RE(XW_11_O_39_)_2_] building blocks in a series of organic–inorganic hybrids based on lacunary Keggin silico- and germanotungstates [160,161,162,163,164].

Dap, like en segments, is a bidentate ligand that has been used in PBTREHDs crystal structures [165,166,167,168,169]. A behavioral study showed that copper dap complexes generate 1D “dendritic” chain-like TM-Ln heterometallic POMs, whereas en generated a 2D TM-Ln heterometallic sheet architecture [157]. In 2014, a series of 1D antiparallel Cu^II^-RE^III^ heterometallic germanotungstate polymeric chains were reported in which [Ln(H_2_O)_3_(α-GeW_11_O_39_)]^5−^ moieties were first connected with each other and then these 1D chains were linked together through bridging [Cu(dap)_2_]^2+^ cations and formed a 1D double-chain architecture [170]. In a similar case, an eight-coordinate RE ion with distorted square antiprism geometry occupy the vacant site of the [*α*-SiW_11_O_39_]^8−^ polyanion and one free space is coordinated to adjacent polyanions, while two [*α*-H_2_SiW_11_O_39_RE(H_2_O)_3_] (RE = Ce^III^, Pr^III^, Nd^III^, Sm^III^, Eu^III^, Gd^III^, Tb^III^, Dy^III^, Er^III^) units are connected to adjacent fragments by Cu(dap)_2_ linkage. There are [Cu(dap)_2_(H_2_O)] complexes connected to the surface oxygen of polyanions that, by incorporating hydrogen bonding, established 3D architecture [171]. 

In a recent study by Wu et al., a rare coordination mode was reported for phen ligands. It was observed that phen ligands preferred Ln over TM. The authors attributed this to the steric hindrance effect in the structure of (Hphen)_2_[Fe(phen)_3_]_2_[Dy(phen)Fe(B-*α*-GeW_9_O_34_)]_2_ [172]. This ligand can generate π–π stacking interactions which help to stabilize the structure [173]. 

In the 1D chain of [Cu_2_(tpy)_2_][RE(H_2_O)_3_K(*α*-HSiW_11_O_39_)]Cl·2H_2_O (RE = Sm, Eu) compounds, the RE element of {RE-*α*-SiW_11_} cluster is in connection with {Cu/tpy} segments via Cu–O bonds and they expand a 1D circle-connecting-circle chain [174]. Zhou et al. demonstrated the first example of an organic–inorganic hybrid with a 3D framework based on the RE-containing monovacant silicotungstate Keggin-type POMs and bimpy ligands. [Cu_2.5_(bimpy)_2_(H_2_O)_2_][RE(H_2_O)_3_(*α*-SiW_11_O_39_)]·xH_2_O [RE = Eu^III^, Sm^III^, Ho^III^, Y^III^, and Ce^III^] compounds were isostructural, showing a 3D framework featuring {–SiW_11_–RE–SiW_11_–}_n_ inorganic chains and {Cu/bimpy} metal–organic ribbons, stemmed from Cu^2+^ ions and bimpy ligands. The adjacent {Cu/bimpy} ribbons were connected by two antiparallel {–SiW_11_–Eu–SiW_11_–}_n_ chains and created a 2D sheet structure, which was further extended to a 3D framework [175].

**Figure 4:**
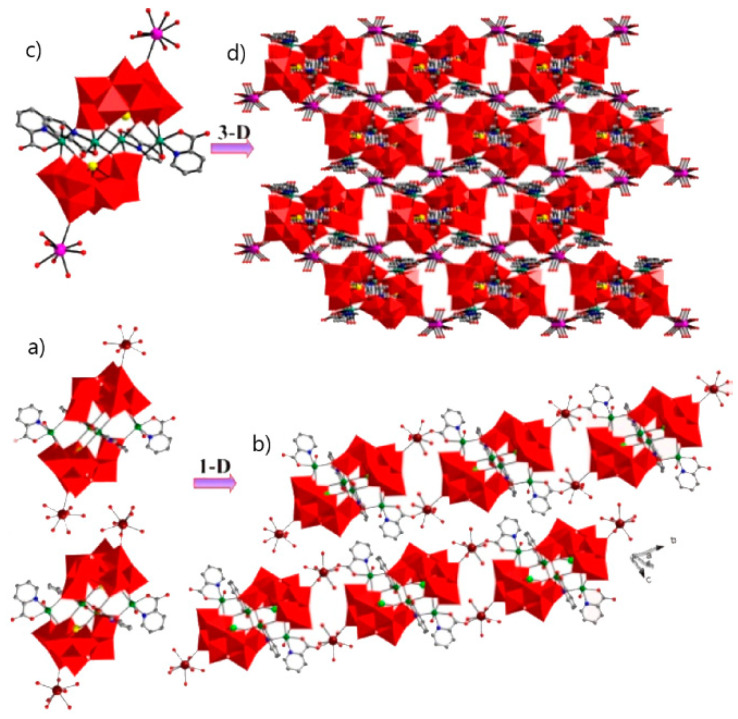
(**a**) View of the pic-substituted Krebs-type unit observed in {[RE(H_2_O)_6_]_2_[Fe_4_(H_2_O)_2_(Hpic)_2_(pic)_2_(B-β-SbW_9_O_33_)_2_]}_2_ (RE = Ho^III^, Er^III^). (**b**) One-D double chain observed in previous compounds. (**c**) View of the pic-substituted Krebs-type unit observed in [RE(H_2_O)_5_]2[Fe_4_(H_2_O)_2_(pic)_4_(B-β-SbW_9_O_33_)_2_] [RE = La^III^, Pr^III^, Nd^III^, Sm^III^, Eu^III^] compounds. (**d**) Three-D extended framework observed in these structures [179].

The substitution of two water molecules by two thr ligands in the [Fe_4_(H_2_O)_10_(B-*β*-SbW_9_O_33_)_2_]^6−^ polyanion [176] led to the formation of [RE(H_2_O)_8_]_2_[Fe_4_(H_2_O)_8_(thr)_2_][B-*β*-SbW_9_O_33_]_2_·22H_2_O (RE = Pr^III^, Nd^III^, Sm^III^, Eu^III^, Gd^III^, Dy^III^, Lu^III^) inorganic–organic hybrids in which two nona-coordinated RE centers were attached to terminal oxygen of two {B-*β*-SbW_9_O_33_} subunits [177]. Similar architecture was achieved by changing heteropoly to {B-*β*-AsW_9_O_33_} subunits [178]. Hpic is another interesting ligand that can generate diverse coordination modes within different coordination geometries of RE ions. The presence of middle RE centers, Gd^III^, and Dy^III^ ions in the solution containing Hpic, Fe^III^ ion, and {B-*α*-SbW_9_} precursor produced discrete structures with different architectures. The molecular structure of these compounds consisted of two types of non-Krebs-type quadripic-inserted [Fe_2_W_4_O_9_(H_2_O)_2_(Hpic)_4_(B-*β*-SbW_9_O_33_)_2_]^6−^ and {[RE(H_2_O)_8_]_2_[Fe_4_W_2_O_7_(H_2_O)_4_(pic)_2_(Hpic)_2_(B-*β*-SbW_9_O_33_)_2_]}^4−^ moieties. In the first subunit hexa-nuclear {Fe_2_W_4_O_9_(H_2_O)_2_(Hpic)_4_} group and the second subunits, the octa-nuclear {[Dy(H_2_O)_8_]_2_[Fe_4_W_2_O_7_(H_2_O)_4_(pic)_2_(Hpic)_2_} group was sandwiched by two {B-*β*-SbW_9_O_33_} polyanions. In the case of Ho^III^, Er^III^ metal ions eight aqua ligands were substituted by four Hpic ligands (Figure 4a). Although both sides of this polyanion were supported by two [RE(H_2_O)_5_]^III^ groups, the structure was extended to a 1D heterometallic double chain through the connection of the mentioned atoms in the same direction (Figure 4b). Similar structures were also observed for a series of compounds containing La^III^, Pr^III^, Nd^III^, Sm^III^, and Eu^III^ cations, in which the connection of one O atom of a carboxylate moiety and one RE^III^ ion of the adjacent unit and the interconnection of these chains constructed a 3D extended framework (Figure 4d) [179].

Because of high affinity of RE cation linkers to the anionic surface of polyanions, their utilization in synthesis is challenging and almost leads to precipitation [180,181]. By introducing O-donor ligands to the reaction medium Zhao et al. [182] successfully established the 3D K_4_Na_4_[Ce_2_(ox)_3_(H_2_O)_2_]_2_{[Mn(H_2_O)_3_]_2_[Mn_4_(GeW_9_O_34_)_2_(H_2_O)_2_]·14H_2_O hybrid with Weakley sandwich-type structure via tetra Mn^II^ belt and 3d-4f connector. Zhang and co-workers prepared two types of RESP hybrids based on phosphotungstates via oxalate linkages. The {[(*α*-PW_11_O_39_)RE(H_2_O)]_2_(ox)}^10−^ (RE = Y^III^, Dy^III^, Ho^III^, and Er^III^) hybrid consisted of seven coordinated REs substituted into mono-lacunary phosphotungstates in which one ox ligand interconnected two polyanion units and generated dimeric architectures. In another work, a 3d-4f heterometallic cluster sandwiched phosphotungstate dimers of [Fe_2_RE(*β*-PW_10_O_37_)_2_(tart)]^9−^ (RE = La^III^, Ce^IIIIII^, Sm^III^, Tb^III^). Fe–O–RE–O–Fe bonds with the support of tart ligands linked [*β*-PW_10_O_37_]^9−^ polyanions and stabilized the structure [183]. 

The employment of two types of TMs in the presence of lanthanoid fragments would improve a special property such as the magnetic susceptibility of POM-based clusters. Accordingly, Sato et al. reported five unique sandwich-type polyanions [FeM_4_{RE(L)_2_}_2_O_2_(A-*α*-SiW_9_O_34_)_2_] (M = Mn^III^, Cu^II^; RE = Gd^III^, Dy^III^, Lu^III^; L = acac, hfac) via a three-step successive introduction of metal ions into tri-lacunary Keggin-type POM [A-*α*-SiW_9_O_34_]^10−^ in the organic solution. The hepta-nuclear cluster consisted of one central Fe^II^ cation surrounded by four pyramid {MnO_5_} moieties between two polyoxoanions [184].

## 5. Inorganic–Organic Hybrids Based on PBTREHD with Mixed Organic Ligands

**Figure 5:**
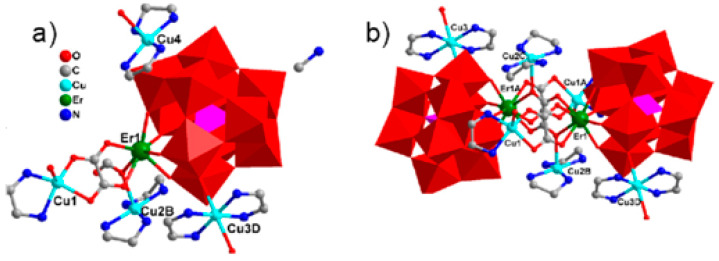
(**a**) Polyhedral and ball-and-stick representation of the asymmetrical unit of {[Cu(en)_2_]_3_[Cu(en)(ox)]_2_[RE_2_(ox)(α-SiW_11_O_39_)_2_]}. (**b**) The ox-bridging dimeric {[Cu(en)_2_]_3_[Cu(en)(ox)]_2_[Er_2_(ox)(α-SiW_11_O_39_)_2_]}^6−^ unit [190].

As mentioned before, O-donor ligands prefer to interact with RE cations in the presence of TM ions; therefore, the introduction of organic fragments containing carboxylate units in the reaction including mono-RE-substituted Keggin polyanions leads to the dinuclear core instead of a cubane moiety [149,185]. Similar dimeric inorganic–organic hybrids based on monolacunary phosphotungstate and germanotungstate polyanions were reported in which a double carboxylate bridging motif coordinated to RE centers that hydrated {Cu(en)_2_} cations balanced the overall charge of constructions [186,187,188]. Du’s group reported the first 1D ladder-like polyanion chain constructed from monovacant Keggin-type polyanions, RE, TM complexes, and two types of organic ligands. In this case, acetate ions bolt two 2:2-type cerium-substituted [*α*-SiW_11_O_39_]^8−^ parts. RE ions in these hybrids were coordinated to tetradentate monolacunary polyoxometalate and three carboxylic O atoms from acetate groups and one water molecule via eight-coordinate square antiprism geometry [185]. In the [Cu(en)_2_(H_2_O)][Cu(en)_2_][Tb(*α*-PW_11_O_39_)(H_2_O)_2_(ox)Cu(en)]∙6H_2_O hybrid, tetradentate ox ligand acted as a bridge between two terbium associated with [*α*-PW_11_O_39_]^7−^ anions or two copper ions and constructed a 1D chain along the c-axis and also coordinated to [Cu(en)_2_] units [189]. A 1D zigzag chain structure was derived from [CeGeW_11_O_39_]^5−^ polyanions by ox and [Cu(en)_2_]^2+^ fragments, alternatively. It is interesting that the ox^−^, as a tetradentate ligand, coordinated to [Cu(en)_2_]^2+^ in addition to its linking of two RE centers [186]. In the case of a 2D {[Cu(en)_2_]_3_[Cu(en)(ox)]_2_[RE_2_(ox)(*α*-SiW_11_O_39_)_2_]} (RE = Er^III^, Sm^III^) sheet, two [RE(*α*-SiW_11_O_39_)_2_] subunits were connected by one free ox moiety and two ox fragments of {Cu(en)(ox)} complexes and constructed a dimeric structure (Figure 5b). Two [Cu(en)(ox)] groups linked two [Cu_2_RE_2_(ox)(SiW_11_)_2_] (Figure 5a) as double chain and one [Cu_3_(en)_2_]^2+^ bridge linked this dimeric structure that was further interconnected to generate a 2D layer [190].

Zhang et al. separated a family of organic–inorganic hybrids based on silicotungstate derivatives with RE-TM heterometals and 2,6-pzdc and en mixed ligands. 2,6-pzdc and its protonated form have various N/O donor agents that, upon participating in hydrogen bonding, facilitate the construction of supramolecular structures [191]. In {[Cu(en)_2_]_2_[Cu(2,3-pzdc)_2_][(*α*-H_2_SiW_11_O_39_)Ce(H_2_O)]_2_}^2+^ hybrids, two carboxylate moieties of two 2,3-pzdc subunits acted as linkage and adjoined cerium centers to construct 1D double-chain architecture. Adjacent N and O atoms of the 2,3-pzdc were linked to Cu^II^ coordinated to the O atom of a polyanion and the last free O atoms of a pentadentate organic ligand trapped by cerium. Ultimately, [Cu(en)_2_]^2+^ was linked to the surface of inorganic ligands and thus, hydrogen bonding 3D framework was obtained [192]. Aromatic N-donor organic ligands not only incorporate the coordination sphere of copper ions but also reinforce the fortification of building blocks by weak interactions [193,194]. Cao’s group demonstrated that the interaction between the [RE(PW_11_O_39_)_2_]^11−^ (RE = La, Pr, Eu, Gd, Yb) cluster and dinuclear copper(II)-ox complexes [Cu_2_(bpy)_2_(*µ*-ox)]^2+^ leads to a 1D infinite chain that further extends to 3D nets via π–π interactions of bipyridine rings of adjacent chains [195]. Niu et al. reported organic–inorganic hybrids composed of sandwich-type [RE(*α*-PW_11_O_39_)_2_]^11−^ building blocks and a copper complex with two different types of N-donor organic ligands as linkage. When [RE(*α*-PW_11_O_39_)_2_] (RE = Ce^III^, Pr^III^) dimers were connected by [Cu(en)(2,2′-bipy)]^2+^ and [Cu(en)_2_]^2+^, a 1D zigzag chain was formed whilst strong hydrogen bonding constructed a 2D lattice [196]. Another interesting example was observed in a series of some isostructural [Cu(cyclam)]_2_[{Cu(cyclam)}_4_{(*α*-GeW_11_O_39_)RE(H_2_O)(OAc)}_2_]·18H_2_O (RE = La−Lu) hybrids where acetate ions connected two {(*α*-GeW_11_O_39_)RE} polyanions, while three type {Cu(cyclam)}^2+^ moieties had different structural roles: surface-appended antenna unit, linking moieties, and charge-balancing cation [197].

**Figure 6:**
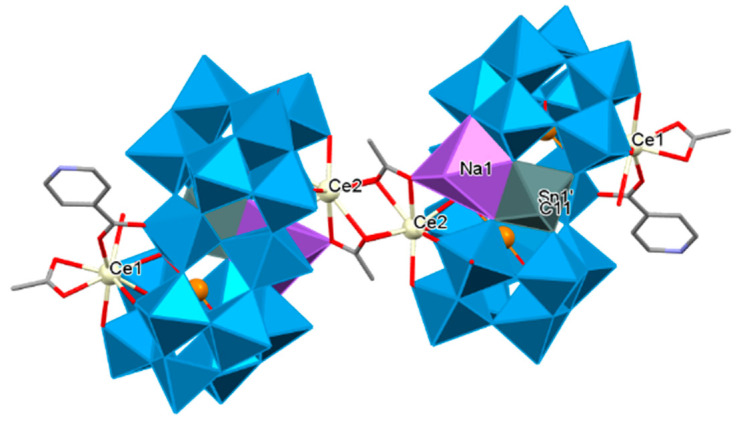
The dimeric skeleton of Na_2_{[Sn(CH_3_)W_2_O_4_(IN)][(B-*α*-TeW_8_O_31_)Ce(H_2_O)(OAc)]_2_}_2_ [198].

The first inorganic–organic hybrid of RE-substituted tellurotungstates containing three different organic ligands dimethylamine hydrochloride, acetic acid, and IN was reported by Han’s group. Accordingly, a family of unique tellurotungstate-based organotin-RE heterometallic hybrids [H_2_N(CH_3_)_2_]_6_H_12_Na_2_{[Sn(CH_3_)W_2_O_4_(IN)][(B-*α*-TeW_8_O_31_)RE(H_2_O)(OAc)]_2_}_2_·25H_2_O [RE = Ce^III^, Pr^III^, Nd^III^, Sm^III^, Eu^III^, Gd^III^, Tb^III^] were synthesized. In these structures, two dimeric {[Sn(CH_3_)W_2_O_4_(IN)][(B-*α*-TeW_8_O_31_)Nd(H_2_O)(OAc)]}^10−^ units were linked by two OAc^−^ connectors. Two rare tetravacant [B-*α*-TeW_8_O_31_]^10−^ subunits sandwiched a cap-sharing IN-decorated [W_2_O_4_(IN)]^3+^ fragment. One [Sn(CH_3_)]^3+^ group via penta-coordinate square pyramid configuration connected to O atoms of the equatorial belt W centers and eventually, two crystallographically independent RE ions occupied vacant sites of the tetralacunary [B-*α*-TeW_8_O_31_]^10−^ subunits via nonacoordinate severely distorted tricapped trigonal prism geometry to construct the new territory of an inorganic–organic hybrid (Figure 6) [198].

[H_2_N(CH_3_)_2_]_8_K_2_Na_4_[RE_2_(OAc)_2_(H_2_O)_4_Fe_2_(2,5-pdc)_2_(B-*β*-TeW_9_O_33_)_2_][RE_2_(H_2_O)_8_Fe_2_(2,5-pdc)_2_(B-*β*-TeW_9_O_33_)_2_]·50H_2_O [RE = Eu^III^, Tb^III^, Dy^III^, Er^III^; 2,5-pdc = 2,5-pyridinedicarboxylic acid] isostructural compounds, owing to the unusual decoration of heterometallic centers by various organic components in the field of POM-based TM−RE heterometallic derivatives, have fascinating structures. On the other hand, two asymmetric sandwich-type subunits, [RE_2_(OAc)_2_(H_2_O)_4_Fe_2_(2,5-pdc)_2_(B-*β*-TeW_9_O_33_)_2_]^8−^ and [RE_2_(H_2_O)_8_Fe_2_(2,5-pdc)_2_(B-*β*-TeW_9_O_33_)_2_]^6−^, can be regarded as classic Krebs-type [Fe_4_(H_2_O)_10_(*β*-TeW_9_O_33_)_2_]^4−^ fragments in which two external Fe^III^ ions are substituted by two RE complexes. Among the two O-donor ligands in the structure, only 2,5-pdc as a linkage, incorporating N and O atoms, conduce tetrameric and then 1D chain arrangement [199].

## 6. Conclusions

The localized surface charge and diverse structures of LPOMs have made them reactive building blocks for the construction of inorganic-organic architectures. The structures of the lacuna itself as well as the main coordination modes of organic fragments are key parameters that need to be considered. The topology of the final hybrid depends on the number of vacancies generated in the parent structure. Organic fragments, on the other hand, can work as ornaments, bridges, and stabilizers in the whole structure. Since oxo groups in POM surfaces can only bond with limited species of transition metals, the utilization of organic ligands is crucial. In the present work, we highlighted the most important reports of inorganic–organic hybrid structures combining LPOMs with TM and Ln ions and compared the different roles of various ligands in their interaction with relevant Keggin-type POMs. Our results demonstrate a great variety of organic ligands and their important roles in the architecture of metal-substituted POMs.

## Figures and Tables

**Table 1:** Summary of inorganic–organic hybrids based on TMSPs with N-/O-donor ligands.

Compound	LPOM	Metal	Ligand	Ref.
[(CH_3_)_4_N]_3_[α-PW_11_O_39_{*cis*-Pt(NH_3_)_2_}_2_]⋅10H_2_O [(CH_3_)_4_N]_4_[α-SiW_11_O_39_{*cis*-Pt(NH_3_)_2_}_2_]·13H_2_O [(CH_3_)_4_N]_4_[α-GeW_11_O_39_{*cis*-Pt(NH_3_)_2_}_2_]·11H_2_O	mono	Pt	amine	[42]
[(CH_3_)_4_N]_4_H[α-AlW_11_O_39_{*cis*-Pt(NH_3_)_2_}_2_]⋅11H_2_O [(CH_3_)_4_N]_4_H[α-BW_11_O_39_{*cis*-Pt(NH_3_)_2_}_2_]⋅9H_2_O Cs_4_[α-GeW_11_O_39_{Pt(bpy)}_2_]⋅10H_2_O Cs_3.5_H_0.5_[α-GeW_11_O_39_{Pt(phen)}_2_]⋅3H_2_O	mono	Pt	amine 2,2′-bipyridine 1,10-phenanthroline	[43]
[(CH_3_)_4_N]_4_H[α-PW_11_O_39_{cis-Pt^II^(Me_2_ppz)}]·5H_2_O	mono	Pt	*N,N′*-dimethylpiperazine	[44]
KRb_5_[(PW_10_O_37_)(Ni(H_2_O))_2_(*μ*-1,1-N_3_)]·19H_2_O	di	Ni	azide	[45]
[Ni_6_(*μ*_3_-OH)_3_(en)_3_(H_2_O)_6_(B-*α*-AsW_9_O_34_)]‧6H_2_O [{Ni_7_(*μ*_3_-OH)_3_O_2_(dap)_3_(H_2_O)_6_}(B-*α*-PW_9_O_34_)][{Ni_6_(*μ*_3_-OH)_3_(dap)_3_(H_2_O)_6_} (B-*α*-PW_9_O_34_)][Ni(dap)_2_(H_2_O)_2_]·4.5H_2_O	tri	Ni	ethylenediamine 1,2-diaminopropane	[46]
[Ni_6_(*μ*_3_-OH)_3_(en)_3_(H_2_O)_6_(B-*α*-AsW_9_O_34_)]‧10H_2_O	tri	Ni	ethylenediamine	[47]
(H_2_en)_2_[Cu^II^_8_(en)_4_(H_2_O)_2_(B-*α*-GeW_9_O_34_)_2_]‧5H_2_O (H_2_en)_2_[Cu^II^_8_(en)_4_(H_2_O)_2_(B-*α*-SiW_9_O_34_)_2_]‧8H_2_O [Cu^II^(H_2_O)_2_]_2_[Cu^II^_8_(en)_4_(H_2_O)_2_(B-*α*-SiW_9_O_34_)_2_] [Cu(dap)(H_2_O)_3_]_2_[{Cu_8_(dap)_4_(H_2_O)_2_}(B-*α*-SiW_9_O_34_)_2_]·6H_2_O [Cu^II^(H_2_O)_2_]H_2_[Cu^II^_8_(dap)_4_(H_2_O)_2_(B-α-GeW_9_O_34_)_2_] [Cu^I^(2,2′-bpy)(4,4′-bpy)]_2_[{Cu^I^_2_(2,2′-bpy)_2_(4,4′-bpy)]_2_[Cu^I^_2_Cu^II^_6_(2,2′-bpy)_2_(4,4′-bpy)_2_(B-*α*-GeW_9_O_34_)_2_}]‧2H_2_O	tri	Cu	ethylenediamine 1,2-diaminopropane 2,2′-bipyridine 4,4′-bipyridine	[48]
[H_2_en]_5_[(B-α-SiW_9_O_34_)_2_Mn^III^_4_Mn^II^_2_O_4_(H_2_O)_2_(Hen)_2_]·8H_2_O [H_2_ppz]_4_[H_2_1,3-dap]_2_[(B-α-SiW_9_O_34_)_2_Mn^III^_4_Mn^II^_2_O_4_(H_2_O)_2_(ppz)_2_]·6H_2_O H_2_[H_2_ppz]_4_[(B-α-SiW_9_O_34_)_2_Mn^III^_4_Mn^II^_2_O_4_(H_2_O)_2_(Hppz)_2_]·8H_2_O	tri	Mn	ethylenediamine piperazine 1,3-diaminopropane	[49]
H_10_K_2_Na_2_[Zr_4_(*μ*_3_-O)_2_(*μ*-OH)_2_(en)_2_(B-*α*-GeW_10_O_37_)_2_]‧4DMF‧22H_2_O H_10_K_4_[Zr_4_(*μ*_3_-O)_2_(*μ*-OH)_2_(dap)_2_(B-*α*-GeW_10_O_37_)_2_]‧6DMF‧18H_2_O	di	Zr	ethylenediamine 1,2-diaminopropane	[50]
[Cu(en)_2_(H_2_O)]_2_{[Cu(en)_2_][*α*-PCuW_11_O_39_Cl]}‧3H_2_O {[Cu(en)_2_(H_2_O)][Cu(en)_2_]_2_[*α*-PCuW_11_O_39_Cl]}‧6H_2_O [{Cu(en)_2_}_3_(*α*-PCuW_11_O_39_Cl)]‧6H_2_O	mono	Cu	ethylenediamine	[51]
{[Cu(en)_2_(H_2_O)]_2_[Cu(en)_2_][*α*-XCuW_11_O_39_]}‧5H_2_O (X = Si^IV^/Ge^IV^), {[Cu(deta)(H_2_O)_2_]_2_[Cu(deta)(H_2_O)][*α*-XCuW_11_O_39_]}‧5H_2_O (X = Ge^IV^/Si^IV^)	mono	Cu	ethylenediamine diethylenetriamine	[52]
[Cu(en)_2_(H_2_O)]_2_[Cu(en)_2_][Cu_6_(en)_2_(H_2_O)_2_(B-α-AsW_9_O_34_)_2_]·en·9H_2_O [Cu(dap)_2_]_3_[Cu_6_(dap)_2_(H_2_O)_2_(B-α-AsW_9_O_34_)_2_]·4H_2_O	tri	Cu	ethylenediamine 1,2-diaminopropane	[53]
[Cu(en)_2_]{[Cu_2_(en)_2_(*μ*-1,1-N_3_)_2_(H_2_O)]_2_[Cu_6_(en)_2_(H_2_O)_2_(B-α-PW_9_O_34_)_2_]}·6H_2_O	tri	Cu	ethylenediamine	[54]
[Cu(dap)_2_]_2_{[Cu(dap)_2_(H_2_O)]_2_[Cu_6_(dap)_2_(B-*α*-SiW_9_O_34_)_2_]}‧4H_2_O	tri	Cu	1,2-diaminopropane	[58]
[Cu(dien)(H_2_O)]_2_{[Cu(dien)(H_2_O)]_2_[Cu(dien)(H_2_O)_2_]_2_[Cu_4_(SiW_9_O_34_)_2_]}⋅5H_2_O [Zn(dap)_2_(H_2_O)]_2_{[Zn(dap)_2_]_2_[Zn_4_(Hdap)_2_(PW_9_O_34_)_2_]}⋅8H_2_O [Zn(dap)_2_(H_2_O)]_4_[Zn(dap)_2_]_2_{(dap)_2_{[Zn(dap)_2_]_2_[Zn_4_(HSiW_9_O_34_)_2_]}{[Zn(dap)_2_(H_2_O)]_2_[Zn_4_(HSiW_9_O_34_)_2_]}}⋅13H_2_O	tri	Cu, Zn	diethylenetriamine 1,2-diaminopropane	[59]
[Cu(H_2_O)_2_]H_2_[Cu_8_(dap)_4_(H_2_O)_2_(*α*-B-GeW_9_O_34_)_2_]	tri	Cu	1,2-diaminopropane	[60]
{[Co(dap)_2_(H_2_O)]_2_[Co(dap)_2_]_2_[Co_4_(Hdap)_2_(*B*-*α*-HGeW_9_O_34_)_2_]}·7H_2_O	tri	Co	1,2-diaminopropane	[61]
[Ni_6_(*μ*_3_-OH)_3_(H_2_O)_2_(dien)_3_(B-*α*-PW_9_O_34_)].4H_2_O [Ni_6_(*μ*_3_-OH)_3_(H_2_O)_6_(dap)_3_(B-*α*-XW_9_O_34_)] (X = Si, P)	tri	Ni	diethylenetriamine diaminopropane	[62]
[Cu(dien)(H_2_O)]_2_[Cu_2_(dien)_2_]_2_H_2_[Bi_2_W_20_O_70_]‧15H_2_O	tri	Cu	diethylenetriamine	[63]
[Cu_2_(phen)_2_(*μ*-ox)]{[Cu(phen)(H_2_O)_2_][Cu_4_ (H_2_O)_4_Cu_2_(phen)_2_(AsW_9_O_33_)_2_]}·6H_2_O	tri	Cu	oxalate 1,10-phenanthroline	[65]
[Cu(bpy)(H_2_O)][H_2_PW_11_O_39_Cu_2_(bpy)_2_(H_2_O)(OH)]·1.5H_2_O	mono	Cu	2,2′-bipyridine	[66]
[Cu^I^_3_(phen)_3_Cl_2_][Cu^II^(phen)_2_][Cu^II^_2_(phen)_2_(*α*-PW_11_O_39_)] (Cuphen)_2_[Cu(phen)_2_]_2_[Cu_6_phen_2_(GeW_9_O_34_)_2_]∙2H_2_O	tri	Cu	1,10-phenanthroline	[67]
[{Cu^I^_3_(phen)}_3_Cl_2_]{[Cu^II^(phen)_2_][Cu^II^_2_(phen)]}_2_(*α*-PW_11_O_39_) Na[Cu^II^_2_(bpy)_2_(OH)_2_][Cu^II^_2_(bpy)_2_(H_2_O)(*α*-PW_11_O_39_)]·3H_2_O [Cu^II^(bpy)(H_2_O)][Cu^II^_2_(bpy)_2_(H_2_O)(*α*-HPW_11_O_39_)]·H_2_O	mono	Cu	2,2′-bipyridine 1,10-phenanthroline	[68]
[H_2_AsW_11_O_39_][Cu_3_(bpy)_3_(H_2_O)_2_(OH)]·2H_2_O	mono	Cu	2,2′-bipyridine	[69]
[Cu^I^(H_2_O)(Hbpp)_2_]⊂{[Cu^I^(bpp)]_2_[PW_11_Cu^II^O_39_]}	mono	Cu	1,3-bis(4-pyridyl)propane	[70]
[{Si_2_W_22_Cu_2_O_78_(H_2_O)}{Cu_2_(phen)_2_(H_2_O)(ac)_2_}_2_]^8−^ [{SiW_11_O_39_Cu(H_2_O)}{Cu_2_(phen)_2_(H_2_O)(ac)_2_}]^4−^	mono	Cu	acetate 1,10-phenanthroline	[71]
[Cu^I^_3_(pz)_2_(phen)_3_]_2_[Cu^I^(phen)_2_][{Na(H_2_O)_2_}{(V^IV^_5_Cu^II^O_6_)(As^III^W_9_O_33_)_2_}]·6H_2_O	tri	V, Cu	1,10-phenanthroline pyrazine	[72]
[{Na(H_2_O)_2_}_3_{M(imi)}_3_(SbW_9_O_33_)_2_]^9−^	tri	M = Co, Ni, Zn, Mn	imidazole	[73]
[{K(H_2_O)_0.5_}_2_{K_2_(H_2_O)_3_}{Ni(H_2_O)(en)_2_}_2_{Ni_4_(H_2_O)_2_(PW_9_O_34_)_2_}] [Cu_6_(Himi)_6_{As^III^W_9_O_33_}_2_]·5H_2_O (H_2_btp)_4_[Fe^III^_2_Fe^II^_2_(H_2_O)_2_(AsW_9_ O_34_)_2_]·4H_2_O	tri	Ni, Cu, Fe	ethanediamine imidazole 1,3-bis(1, 2, 4-triazol-1-yl) propane	[74]
[Na(H_2_O)_2_]_3_[C_3_H_5_N_2_]_2_[SbW_9_O_33_]_2_[Fe^II^(C_3_H_4_N_2_)]_3_·4H_2_O	tri	Fe	imidazole	[75]
{Na_0.7_M_5.3_(H_2_O)_2_(imi)_2_(Himi)(SbW_9_O_33_)_2_}^6−^	tri	Ni, Co	imidazole	[76]
[M_6_(imi)_6_(B-*α*-AsW_9_O_33_)_2_]^6−^	tri	Mn, Ni, Zn, Cu	imidazole	[77]
{[Ag_7_(H_2_biim)_5_][PW_11_O_39_]}·Cl·H_3_O	mono	Ag	2,2′-Biimidazole	[80]
{[Cu_8_(tz)_8_(Htz)_4_(H_2_O)_5_][PMo^VI^_10_Mo^V^O_39_]}∙10H_2_O	mono	Cu	1H-tetrazole	[84]
K_8_[(OC)_3_Mn(A-*α*-H_2_GeW_9_O_34_)]·10H_2_O K_8_[(OC)_3_Mn(A-α-H_2_SiW_9_O_34_)]·11H_2_O	tri	Mn	carbonyl	[85]
(YOH_2_)_3_(CO_3_)(A-*α*-PW_9_O_34_)_2_^11−^	tri	Y	carbonate	[86]
(NH_4_)_3_H_5_[{Mn_4_(H_2_O)_10_}(*β*-BiW_9_O_33_)_2_{Mn(CO)_3_}_2_]·31H_2_O Na_6_[(CH_3_)_4_N]_2_[{Mn_4_(H_2_O)_10_}(*β*-SbW_9_O_33_)_2_{Mn(CO)_3_}_2_]·36H_2_O [(CH_3_)_4_N]_2_{Mn(H_2_O)_6_}_2_[{Mn_3.5_W_0.5_(H_2_O)_10_}(*β*-SbW_9_O_33_)_2_{Mn(CO)_3_}_2_]·12H_2_O [(CH_3_)_4_N]_2_{Mn(H_2_O)_6_}_2_[{Mn_3.5_W_0.5_(H_2_O)_10_}(*β*-BiW_9_O_33_)_2_{Mn(CO)_3_}_2_]·12H_2_O	tri	Mn	carbonyl	[87]
Na_2_H_2_[(CH_3_)_4_N]_6_[Te_2_W_20_O_70_{Re(CO)_3_}_2_]·20H_2_O	tri	Re	carbonyl	[88]
[*γ*-H_2_SiW_10_O_36_Pd_2_(OAc)_2_]^4−^	di	Pd	acetate	[89]
K_4_H_6_[Zr_4_(OH)_6_(CH_3_COO)_2_(α-PW_10_O_37_)_2_]·23H_2_O	di	Zr	acetate	[90]
[H_2_N(CH_3_)_2_]_14_[As_4_W_40_O_140_{Ru_2_(CH_3_COO)}_2_]⋅22 H_2_O	tri	Ru	acetate	[91]
Cs_6_Na_5_[Zr_6_O_4_(OH)_4_(H_2_O)_2_(CH_3_COO)_5_(AsW_9_O_33_)_2_]·80H_2_O Cs_6_Na_5_[Hf_6_O_4_(OH)_4_(H_2_O)_2_(CH_3_COO)_5_(AsW_9_O_33_)_2_]·80H_2_O	tri	Zr, Hf	acetate	[92]
Na_15_[(Mn^II^(COOH))_3_(AsW_9_O_33_)_2_]‧15H_2_O	tri	Mn	acetate	[93]
H[Ni_0.5_(en)(H_2_O)][Ni_6_(en)_3_(OAc)_2_(Tris)(H_2_O)_2_(B-*α*-PW_9_O_34_)]·5H_2_O [Ni(en)_2_][Ni_6_(en)_3_(*μ*_3_-OH)_3_(1,3-bdc)(H_2_O)_2_(B-*α*-PW_9_O_34_)]·9H_2_O [Ni(en)(H_2_O)_4_]_3_[Ni_6_(en)_3_(Tris)(1,3-bdc)_1.5_(H_2_O)_2_(B-*α*-PW_9_O_34_)]·8H_2_O	tri	Ni	ethylenediamine acetate tris(hydroxymethyl)aminomethane 1,3-benzenedicarboxylate	[94]
K_2_H_10_[Zr_4_(H_2_O)_2_(μ-OH)(μ_3_-O)_2_(D-tartH)(GeW_10_O_37_)_2_]·27H_2_O K_2_H_10_[Zr_4_(H_2_O)_2_(μ_3_-O)_2_(gly)_2_(GeW_10_O_37_)_2_]·32H_2_O	di	Zr	D-tartaric acid glycolic acid	[95]
Na_1.5_K_2.5_[Ni(H_2_O)_6_]_0.5_[(SiW_9_O_34_)(OH)_3_Ni_4_(C_6_H_13_NO_2_)_3_]·17H_2_O Na_2_K_12_[Ni(H_2_O)_6_][(SiW_9_O_34_)_2_(OH)_6_Ni_8_(C_6_H_8_O_4_)_3_]·40H_2_O Na_6_K_8_[(SiW_9_O_34_)_2_(OH)_6_Ni_8_(C_10_H_8_O_4_)_3_]·45H_2_O	tri	Ni	dimethylaminobutyric acid adipic acid *p*-phenylenediacetic acid	[96]
Na_2_H_4_{[Mn(H_2_O)_3_]_3_[Mn(H_2_O)_2_]_2_[Mn(H_2_O)][Mn(C_2_O_4_)]_3_[B-*α*-SbW_9_O_33_]_2_}·31H_2_O	tri	Mn	oxalic acid	[97]
TBA_4_[*γ*-SiTi_2_W_10_O_36_(*μ*-OH)_2_(*μ*-BINOLate)]	di	Ti	(R)/(S)-1,1′-bi-2-naphthol	[99]
Na_7_(NH_4_)_5_[{Zr_4_(OH)_6_(OAc)_2_}(SiW_10_O_37_)_2_]·20H_2_O	di	Zr	acetate	[102]
(NH_4_)_4_(TMA)_4_[Zr_4_(μ_3_-O)_2_(L-/D-mal)_2_(B-*α*-HSiW_10_O_37_)_2_] (NH_4_)_4_(TMA)_4_[Zr_4_(μ_3_-O)_2_(L-/D-mal)_2_(B-*α*-PW_10_O_37_)_2_]	di	Zr	L/D malate	[103]
{[Cu_5_(2,2′-bpy)_5_(H_2_O)][GeW_9_O_34_]}_2_‧7H_2_O	tri	Cu	2,2′-bipyridine	[104]
Na[{Cu(2,2′-bpy)(imi)}{Cu(2,2′-bpy)}_2_AsW_9_O_33_As(OH)]‧8H_2_O	tri	Cu	2,2′-bipyridine imidazole	[105]
Na(H_2_O)_6_][Co_3_(OH)(pydz)_4_(H_2_O)_7_][Co_6_(PW_10_O_37_)_2_(pydz)_4_(H_2_O)_6_]·43H_2_O	di	Co	pyridazine	[106]
H_8_K_3_Na_5_[Zr_6_(μ_3_-O)_3_(OH)_3_(OAc)(H_2_O)(*β*-GeW_10_O_37_)_3_]·20H_2_O H_6_K_4_Na_12_[{Zr_5_(μ_3_-OH)_4_(OH)_2_}@{Zr_2_(OAc)_2_(*α*-GeW_10_O_38_)_2_}_2_]·22H_2_O H_4_Na_2_[Na_6_(H_2_O)_22_][Zr_4_(*μ*_3_-O)_2_(OH)_2_(OAc)_2_(*α*-GeW_10_O_37_)_2_]·32H_2_O	di	Zr	acetate	[107]
(NH_4_)_3_Na_5_K_6_[Zr_4_(*μ*_3_-O)_2_(*μ*-OH)_2_(ox)_2_(SiW_10_O_37_)_2_]·23H_2_O	di	Zr	oxalate	[108]
Na_2_H_4_[Fe_4_(H_2_O)_8_(3, 4-pdc)_2_(B-*β*-SbW_9_O_33_)_2_]·40H_2_O	tri	Fe	pyridine-3, 4-dicarboxylic acid	[109]

**Table 2:** List of bridging ligands and their characterization in the dimeric inorganic–organic hybrids based on RESPs.

Ligand	RE···RE Distance (Å)	RE-O_carboxylate_ (Å)	RE-O_POM_	Compound	Ref
Acetic acid	4.085	2.418, 2.450	2.259–2.290	[{Yb(*α*-SiW_11_O_39_)(H_2_O)}_2_(*µ*-OAc)_2_]^12−^	[116]
⸗	4.148	2.460, 2.505	2.319–2.360	[{Eu(*α*-SiW_11_O_39_)(H_2_O)}_2_(*µ*-OAc)_2_]^12−^	[117]
⸗	4.133	2.446, 2.492	2.317–2.349	[{Gd(*α*-SiW_11_O_39_)(H_2_O)}_2_(*µ*-OAc)_2_]^12−^	[117]
⸗	4.111	2.432, 2.472	2.293–2.349	[{Tb(*α*-SiW_11_O_39_)(H_2_O)}_2_(*µ*-OAc)_2_]^12−^	[117]
⸗	4.103	2.436, 2.454	2.290–2.339	[{Dy(*α*-SiW_11_O_39_)(H_2_O)}_2_(*µ*-OAc)_2_]^12−^	[117]
⸗	4.086	2.413, 2.458	2.275–2.315	[{Ho(*α*-SiW_11_O_39_)(H_2_O)}_2_(*µ*-OAc)_2_]^12−^	[117]
⸗	4.070	2.410, 2.432	2.273–2.320	[{Er(*α*-SiW_11_O_39_)(H_2_O)}_2_(*µ*-OAc)_2_]^12−^	[117]
⸗	4.065	2.412, 2.441	2.275–2.291	[{Tm(*α*-SiW_11_O_39_)(H_2_O)}_2_(*µ*-OAc)_2_]^12−^	[117]
⸗	4.082	2.419, 2.437	2.291–2.337	[{Y(*α*-SiW_11_O_39_)(H_2_O)}_2_(*µ*-OAc)_2_]^12−^	[118]
⸗	4.110	2.430, 2.450	2.300–2.351	[{Y(*α*- GeW_11_O_39_)(H_2_O)}_2_(*µ*-OAc)_2_]^12−^	[118]
⸗	4.154	2.502, 2.503, 2.525	2.365–2.404	[{Sm(*α*-PW_11_O_39_)(H_2_O)(η^2^,μ-1,1)-OAc }_2_]^10−^	[119]
⸗	4.131	2.474, 2.486, 2.515	2.334–2.362	[{Eu(*α*-PW_11_O_39_)(H_2_O)(η^2^,μ-1,1)-OAc }_2_]^10−^	[119]
⸗	4.078	2.424, 2.459, 2.475	2.312–2.369	[{Gd(*α*-PW_11_O_39_)(H_2_O)(η^2^,μ-1,1)-OAc }_2_]^10−^	[119]
⸗	4.106	2.446, 2.489, 2.456	2.106–2.359	[{Tb(*α*-PW_11_O_39_)(H_2_O)(η^2^,μ-1,1)-OAc }_2_]^10−^	[119]
⸗	4.068	2.437, 2.451, 2.453	2.292–2.341	[{Ho(*α*-PW_11_O_39_)(H_2_O)(η^2^,μ-1,1)-OAc}_2_]^10−^	[119]
⸗	4.121	2.462, 2.482, 2.485	2.327–2.384	[{Er(*α*-PW_11_O_39_)(H_2_O)(η^2^,μ-1,1)- OAc }_2_]^10−^	[119]
Oxalic acid	6.163	2.363, 2.402	2.252–2.294	{[(*α*-PW_11_O_39_)Y(H_2_O)]_2_(ox)} ^10−^	[125]
⸗	6.160	2.362, 2.370	2.251–2.291	{[(*α*-PW_11_O_39_)Dy(H_2_O)]_2_(ox)} ^10−^	[125]
⸗	6.136	2.353	2.203–2.286	{[(*α*-PW_11_O_39_)Ho(H_2_O)]_2_(ox)} ^10−^	[125]
⸗	6.161	2.373, 2.409	2.247–2.310	{[(*α*-PW_11_O_39_)Er(H_2_O)]_2_(ox)} ^10−^	[125]
⸗	6.360	2.295, 2.414	1.983–2.143	{(*α*-x-PW_10_O_38_)Tm_2_(ox)(H_2_O)_2_}^3^^−^	[125]

## Data Availability

Data is contained within the article.

## Abbreviations

OAc: ac	Acetate
acac	Acetylacetonate
ala	Alanine
ampH2	(Aminomethyl)phosphonic acid
bdc	Benzenedicarboxylate
bdyl	2,2′-Bipyridinyl
bimpy	3,5-Bis(1-imidazolyl)pyridine
BINOL	(R)- or (S)-1,1′-bi-2-naphthol bipy, bpy: 2,2′-Bipyridine
bpp	1,3-bis(4-pyridyl)propane
btp	1,3-bis(1,2,4-triazol-1-yl) propane
cyclam	1,4,8,11-Tetraazacyclotetradecane
dap	Diaminopropane
dien, deta	Diethylenetriamine
DMAHC	dimethylamine hydrochloride
DMEA	N,N-dimethylethanolamine
DMF	Dimethylformamide
DMSO	Dimethyl sulfoxide
en	Ethylenediamine
gly	Glycine
H2biim	2,2′-Biimidazole
hfac	Hexafluoroacetylacetonate
Hpic	2-picolinic acid
Htz	1H-tetrazole
imi	Imidazole
IN	Isonicotinate
Ln	Lanthanoide
LPOM	Lacunary POM
mal	Malate
Me_2_ppz	*N,N*′-dimethylpiperazine
ox	Oxalate
PBTREHD	POM-based TM-RE heterometallic derivative
pdc	Pyridine dicarboxylate
phen	1,10-Phenanthroline
pic	Picolinate
POM	Polyoxometalate
POMOF	Polyoxometalate open framewok/Polyoxometalate-based metal–organic framework
POT	Polyoxotungstate
ppz	piperazine
pydz	Pyridazine
pz	Pyrazine
pzdc	Pyrazinedicarboxylic acid
RE	Rare earth
RESP	Rare earth-substituted POM
tart	Tartrate
taz	1,2,4-1H-triazole
TBA	Tetrabutylammonium
thr	Threonine
TM	Transition metal
TMA	Tetramethylammonium
TMSP	Transition metal-substituted POM
tpy	2,2′:6′,2″-terpyridine
Tris	Tris(hydroxymethyl)aminomethane
